# How Signaling Molecules Regulate Tumor Microenvironment: Parallels to Wound Repair

**DOI:** 10.3390/molecules22111818

**Published:** 2017-10-26

**Authors:** Peter Gál, Lenka Varinská, Lenka Fáber, Štepán Novák, Pavol Szabo, Petra Mitrengová, Andrej Mirossay, Pavel Mučaji, Karel Smetana

**Affiliations:** 1 Department for Biomedical Research, East-Slovak Institute of Cardiovascular Diseases, Inc., 040 11 Košice, Slovakia; lvarinska@vusch.sk (L.V.); szabopavol@gmail.com (P.S.); 2 Department of Pharmacology, Faculty of Medicine, Pavol Jozef Šafárik University, 040 11 Košice, Slovakia; lenka.faber@yahoo.com (L.F.); andrej.mirossay@upjs.sk (A.M.); 3 Department of Pharmacognosy and Botany, Faculty of Pharmacy, Comenius University, 832 32 Bratislava, Slovakia; peta.mitrengova@gmail.com; 4 Institute of Anatomy, 1st Faculty of Medicine, Charles University, 128 00 Prague, Czech Republic; stepan.novak@lf1.cuni.cz; 5 Department of Otorhinolaryngology and Head and Neck Surgery, 1st Faculty of Medicine, Charles University and University Hospital Motol, 150 06 Prague, Czech Republic; 6 BIOCEV, 252 50 Vestec, Czech Republic

**Keywords:** tissue repair, cancer, stem cell, galectin, cytokine

## Abstract

It is now suggested that the inhibition of biological programs that are associated with the tumor microenvironment may be critical to the diagnostics, prevention and treatment of cancer. On the other hand, a suitable wound microenvironment would accelerate tissue repair and prevent extensive scar formation. In the present review paper, we define key signaling molecules (growth factors, cytokines, chemokines, and galectins) involved in the formation of the tumor microenvironment that decrease overall survival and increase drug resistance in cancer suffering patients. Additional attention will also be given to show whether targeted modulation of these regulators promote tissue regeneration and wound management. Whole-genome transcriptome profiling, in vitro and animal experiments revealed that interleukin 6, interleukin 8, chemokine (C-X-C motif) ligand 1, galectin-1, and selected proteins of the extracellular matrix (e.g., fibronectin) do have similar regulation during wound healing and tumor growth. Published data demonstrate remarkable similarities between the tumor and wound microenvironments. Therefore, tailor made manipulation of cancer stroma can have important therapeutic consequences. Moreover, better understanding of cancer cell-stroma interaction can help to improve wound healing by supporting granulation tissue formation and process of reepithelization of extensive and chronic wounds as well as prevention of hypertrophic scars and formation of keloids.

## 1. Introduction

The main reason of limited efficiency of conservative treatment of advanced malignancies is based on the elimination of quickly proliferating cancer cells [1,2] and/or decrease of tumor invasiveness [3]. However, by using this approach we are not able to attack slowly dividing progenitors and cancer stem cells protected by a niche that is called the tumor microenvironment (TME). Although, a growing body of evidence has revealed that the TME differs distinctly from the corresponding normal tissue stroma, remarkable similarities between the connective tissue reaction in wounds and in tumors have been reported by Harold Dvorak’s article “Tumors: wounds that do not heal” published already 30 years ago [4]. Further studies comparing tissue repair and regeneration with aspects of malignancy revealed that these two process cascades do have even more in common (Table 1) [5,6,7].

However, a healing wound represents an exact opposite biological situation. Lack of stem cells and premature differentiation of stem cells present in wounds extends the healing period and in several cases, may lead to extensive scar formation. Furthermore, chronic wounds are characterized by a deficiency of growth factors, thus an optimal treatment should convert the wound environment from a chronic to an acute state [8]. Therefore, the therapeutic strategy of both pathologies calls for a complex approach including modulation of the wound/tumor microenvironments.

Hence, in this review an attempt was made to identify factors associated with tumor growth and spreading with potential implications in wound healing management. In this aspect, it is well known that fibroblasts play the key role in the formation of tumor stroma and/or granulation tissue [5,9,10,11]. Remarkable is their ability to differentiate into myofibroblasts, which play an important role in wound contraction [12] and significantly modulate biological properties of tumors [11]. Although, fibroblasts secrete several growth factors (e.g., insulin-like growth factor 2, bone morphogenetic protein 4), cytokines (e.g., interleukin 6), chemokines (e.g., chemokine (C-X-C motif) ligand 1 (CXCL1), interleukin 8 (IL-8)) as well as important structural macromolecules (collagen, fibronectin, tenascin) forming the extracellular matrix (ECM), existing anti-tumor therapies are mainly focused on the possibility to modulate the incipient cancer cells which has not resulted in significantly improved survival [2,6].

## 2. Tumor Microenvironment

**Figure 1:**
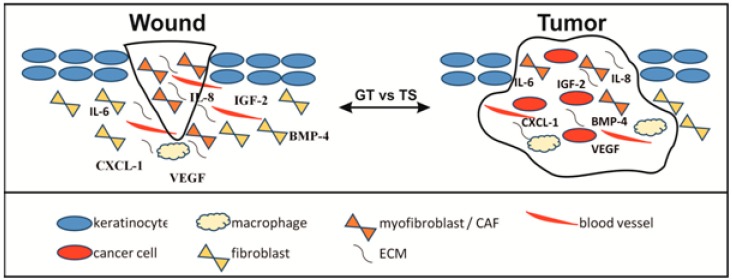
Schematic comparison of wound/tumor microenvironment created by cells located in the granulation tissue/tumor stroma (GT/TS), respectively (interleukin 6/8 (IL-6/-8); insulin-like growth factor 2 (IGF-2); bone morphogenic protein 4 (MBP4); chemokine (C-X-C motif) ligand 1 (CXCL1).

The TME contributes to the development and metastasis of tumors and thus has become a new emerging concept in tumor research and therapeutic strategy (Table 2). TME is composed of cellular (Figure 1) and non-cellular components, i.e., the ECM [13]. Multiple different cell types comprise the cellular compartment of the TME: (i) cells that are present in the normal tissue before tumor development; (ii) cells that are recruited to the tumor-associated stroma from distal sites (i.e., the circulation or bone marrow cells).

The first type is largely comprised of local mesenchyme as one of sources of cancer-associated fibroblasts (CAFs) and endothelial cells (ECs), whereas the second type of cells is largely comprised of immune/inflammatory cells, including T- and B-cells, macrophages, neutrophils, mast cells, and other bone marrow-derived cells including mesenchymal stem cells [7,14]. A growing body of evidence has revealed that the TME differs distinctly from the corresponding normal stroma. Rather than a “bystander”, the TME acts as an active participant in a constant conversation with the tumor. Evidence suggests that there is close link between tumor cells and their TME. Macrophages, CAFs, ECs, and other types of stromal cells control and alter the TME by inducing changes facilitating the tumor cells’ local and distant dissemination. Moreover, these non-neoplastic cells can change their phenotype upon soluble or physical contact-mediated stimulation by tumor cells towards a tumor-promoting one [15,16,17].

Apart from CAFs, ECM matrices induce multiple dynamic interactions with endothelial cells and stimulate the transduction of signals by cross-linking integrin receptors on endothelial cells. Initially viewed as merely a physical barrier, the ECM is now recognized as having a profound effect on the angiogenic phenotype. However, the integrated regulatory mechanism of microvascular endothelial cell response to ECM and angiogenic factors is poorly defined [52,53,54]. Alteration of ECM composition and architecture is a hallmark of tumor stroma and/or wound healing.

## 3. Wound/Keloid Scar Microenvironment and Its Similarity to TME

Wound healing includes an orchestrated cascade of biological processes following injury by which tissue (e.g., skin) repairs itself. This process runs in four basic steps: blood clotting, inflammation, proliferation, and maturation/remodeling. In particular, the proliferation phase of wound healing is accompanied by production of granulation tissue which architecture is very similar to that of a tumor stroma (Figure 1). Here, fibroblasts produce several cytokines/chemokines and growth factors that on the one hand stimulate angiogenesis and on the other hand support the process of reepithelization [55,56,57,58]. Comparing both processes, i.e., cancer growth and wound repair, granulation tissue and tumor stroma have strong supporting roles in maintaining poorly differentiated epithelial cells to proliferate [59]. On the other hand, there exist several differences between tumors and wounds, for instance platelets, which play critical roles in hemostasis seem not to participate to any great extent in the stroma generation of solid tumors [60].

In addition, the normal course of healing can be under not very well understood circumstances terminated by the formation of a pathologic hypertrophic and keloid scars as a result of immature collagen overproduction [61]. However, hypertrophic scars do not extend beyond the initial site of injury and may partially regress over time, keloids extend beyond the original wound area with thicker collagen bundles and do not regress spontaneously [62]. Of note, keloids may in some cases also result in local functional limitations, but mostly represent only a cosmetic issue. In this context it is interesting that some similarities between tumors and scars have been noted [63] including positive role of inflammation supporting micromilieu of keloid origin and progression [64,65]. In this context fibroblast activation protein alpha (FAP-α) and dipeptidyl peptidase IV (DPPIV) are proteases located at the plasma membrane promoting cell invasiveness, tumor growth, and keloid scar formation. It has also been shown that normal adult tissues are generally FAP-α negative. Therefore, inhibiting FAP-α/DPPIV activity may represent a novel way to prevent keloid scaring [66]. From this point of view, targeting fibroblasts, including CAFs, by a monoclonal antibody against FAP (e.g., sibrotuzumab) could have beneficial effects in modulating the TME and in such a way increasing the survival rates of patients. However, clinical trials running in small cell lung cancer and colorectal cancer suffering patients has not resulted in success [67]. Since the tissue distribution of FAP-specific monoclonal antibody was encouraging, investigators have suggested their use as effective vehicles of other therapeutics to the tumor site.

## 4. Roles of Cytokines/Chemokines and the Immune System in the Tumor/Wound Microenvironment Formation

Although, it is now clear that proliferation of cells does not results in formation of tumors, sustained cell proliferation in an environment rich in inflammatory cells, growth factors, activated stroma, and DNA-damage-promoting agents, certainly potentiates and/or promotes the neoplastic risk [68]. Cancer-associated fibroblasts are one of the most abundant stromal cell types in different carcinomas and comprise a heterogeneous cell population. In physiological conditions, normal fibroblasts remain in a quiescent inactive state. CAFs are activated tissue fibrosis stimulating fibroblasts that produce growth factors, cytokines, chemokines and immune modulators [69]. The main source of CAFs seems to be locally residing fibroblast, although they can also be derived from bone marrow mesenchymal cells, pericytes, endothelial cells and smooth muscle cells [12,70]. Of note, CAFs include a phenotypically heterogeneous group of fibroblasts that express, at least, alpha-smooth muscle actin (α-SMA) and vimentin [69]. Furthermore, CAFs secrete a variety of pro-inflammatory factors [71] leading to the recruitment and promotion of immunosuppressive and tumor-promoting immune cells [72], thereby contributing to the establishment of a pro-inflammatory, immune-suppressive, tumor-permissive environment.

Hence, CAFs are a rich source of different secreted factors such as cytokines and chemokines. Therefore, selected inhibitors have been designed to inhibit several pro-inflammatory molecules [6,73,74,75]. Although, the modulation of selected cytokines/chemokines has resulted in interesting findings in several preclinical studies, the therapeutic impact of anti-IL-6 or anti-tumor necrosis factor alpha (TNF-α) monoclonal antibodies [37] demonstrated only limited clinical efficiency when administered separately. For instance, siltuximab monotherapy (IL-6 monoclonal antibody) has appeared to be well tolerated, but its clinical effect is very limited [38]. Wounds in mice lacking IL-6 showed delays in macrophage infiltration, fibrin clearance, and wound contraction that were not seen in mice lacking IL-6 receptor-α alone [76]. Recombinant IL-6 treatment of IL-6 knock-out mice revealed that IL-6 has the ability to induce the expression of transforming growth factor-β1 (TGF-β1) a molecule with emerging importance for tissue fibrosis [77]. Our group for the first time demonstrated that simultaneous blocking of IL-6 and IL-8 is sufficient to fully inhibit CAF-induced human melanoma cell invasiveness [78]. A complex approach to the TME has also revealed that therapeutic targeting of IL-6 and IL-8 receptors using tocilizumab and reparixin significantly decreased metastasis of breast cancer cells to the lungs, liver, and lymph nodes [79]. Even more complex was the approach in the squamous cell carcinoma of the head and neck where a combination of three targets, i.e., IL-6, IL-8 and CXCL1, has been shown to be effective in ameliorating of the TME [6].

Other example has been the antibody against MIF (macrophage migration inhibitory factor) which inhibited tumor angiogenesis and lymphangiogenesis in mice model of osteosarcoma [80]. While promising, there are also safety concerns regarding to systemic treatment with inhibitors of inflammatory molecules, mainly because of its pleiotropic effects on tissue remodeling, immunomodulation and cancer development. MIF has been identified as the key effector mediating beneficial effects of estrogens on wound healing [81]. However, MIF appears to be able to exert both positive and negative effects and its cell-specific relevancy in wound repair remains still unclear [82].

Numerous preclinical studies indicate that the treatment resistance is also resulted from the cancer related activation of nuclear factor kappa B (NF-κB) [83]. Therefore, selected inhibitors of NF-κB and signal transducer and activator of transcription 3 (STAT3) signaling pathways have been tested to increase the effectiveness of treatment of metastatic prostate cancer [26,84]. Notably, EC-70124 (glycosylated indolocarbazole multikinase inhibitor) had profound effects on the prostate cancer stem cell (CSC) subpopulation both in vitro and in vivo. Thus, EC-70124 is a potent inhibitor of the NF-κB and STAT3 signaling pathways and blocked tumor growth and maintenance of prostate CSCs [26]. However, previous studies have shown that long-term NF-κB inhibition led to several unwanted side effects like neutrophilia, liver damage, and acute inflammation mediated by increased IL-1 secretion [85]. In this context, IL-1 expression has been identified as necessary in facilitating the healing process by protecting an open wound from bacterial infection, but the production of new connective tissue and re-epithelization are minimally affected by the absence of its activity [86].

## 5. Roles of Growth Factors in the Tumor/Wound Microenvironment Formation

Furthermore, CAFs secrete several regulators of angiogenesis, including vascular endothelial growth factor (VEGF), TGF-β, hepatocyte growth factor (HGF), epidermal growth factor (EGF), or fibroblast growth factor (FGF). Among the most potent proangiogenic growth factors belong VEGF, which is up-regulated in many tumors and plays a critical role in tumor stroma. CAFs also express receptors such as platelet-derived growth factor receptor alpha (PDGFRα) and platelet-derived growth factor receptor beta (PDGFRβ) [87,88]. Furthermore, CAFs play an important role in remodeling of the ECM by expressing a wide variety of matrix-components and matrix-remodeling enzymes such as neuron glial antigen (NG2), tenascin C, type I collagen, fibronectin, or matrix metalloproteinase 1/stromelysin-1 [89,90]. Research over the last years has provided a body of evidence that CAFs play an important role in controlling tumor fate. The pro-tumorigenic activity of CAFs includes strong paracrine effects impacting on different cell types present in the tumor. Direct stimulation of cancer cells by CAF-derived signals promotes, e.g., cancer cell proliferation [91], migration, invasion [92], and the adoption of a cancer stem cell phenotype by inducing the epithelial to mesenchymal transition (EMT) [93,94].

In this context, VEGF-A monoclonal antibody (Bevacizumab) was the first anti-angiogenic drug approved by the Food and Drug Administration (FDA) in 2004. Bevacizumab has shown clinical activity in different solid tumor types resulting in approval by the FDA for treatment of metastatic colorectal cancer, non-small cell lung cancer, renal cell carcinoma, glioblastoma multiforme, ovarian cancer and metastatic cervical cancer. Next to VEGF, anti-neoplastic strategies have focused also on blocking tyrosine kinases. In this context, PDGFR, c-KIT, and VEGF receptor (VEGFR) are the most commonly inhibited kinases. The FDA has approved over 19 oral kinase inhibitors for the treatment of malignancies in hematology/oncology [95]. In contrast, it is well known that diabetic skin ulcers are difficult to heal due to reduced levels and/or activity of endogenous growth factors. It has been shown that direct delivery of VEGF and basic fibroblast growth factor (bFGF) at the wound site in a sustained and controllable way has enhanced granulation tissue formation and collagen deposition in diabetic mice [96]. Moreover, treatment with human recombinant PDGF has revealed its efficiency in both acute wounds [97] and lower extremity diabetic ulcers [98]. Similarly, intralesional EGF administration three times a week has been shown effective for treatment of diabetic foot ulcers [99].

It has also been revealed that at different wound repair stages different set of specific cytokines and growth factors are required [100]. However, topically administered growth factors, in particular in chronic wounds, have shown limited success which may be a result of several biological events. Firstly, proteases activated at the injury site are able to degrade both endogenous and exogenous growth factors and other signaling molecules [101]. Secondly, the skin layer surrounding the lesion forms a strong barrier protecting the organism from hydrophilic molecules. Finally, derived molecules are rapidly eliminated by the production of wound exudates [102]. Therefore, higher doses and/or repeated administrations over a longer time periods are inevitable which can lead to serious side effects including carcinogenesis.

## 6. Roles of Galectins in the Tumor/Wound Microenvironment Formation

Carbohydrates, frequently in the form of glycoproteins and/or glycolipids, represent an important component of living organisms. As biopolymers, they are able to storage biological information which can be decoded by specific counterpartners—endogenous lectins [103]. Galectins (Gals) are endogenous lectins with very complex biological effects. Nowadays, it is estimated that there exist at least 15 galectins. Human cells are known to express 12 of these galectins, missing murine Gal-5 and -6 and ruminant Gal-11 and -15. Gals, a family of glycan-binding proteins, are abundantly expressed in tumor microenvironments of different human tumors and in the granulation tissue of healing wounds [11,104,105,106,107].

Gal-1 and Gal-3 are most intensively studied in the context of cancer. The expression of Gal-1 is regulated by hypoxia-inducible factor-1, which plays a vital role in tumor supporting microenvironment [108]. It has been proven that Gal-1 is associated with all stages of cancer cell progression and play a prominent role in both tumor cells and stromal cells present in the tumor microenvironment [5,109]. The role of Gal-1 seems to be complex and pleiotropic and its potential therapeutic modulation must be individualized to concrete patient and cancer [86]. Gals-1/-3 are able to induce anoikis in cells of pancreatic cancer [89,110,111,112]. On the other hand, Gal-1 contributes to the creation of an immunosuppressed microenvironment at sites of tumors and plays an important role in the tumor angiogenesis as well as in the tumor growth and its ability to form metastasis [53,106,113]. In particular, Gal-1 promotes tumor escape from immunity by suppressing T cell-mediated cytotoxic immune response by binding to a number of different targets expressing N- or O-glycans [114]. Cell surface glycoproteins on primary T cells (e.g., CD4, CD7, CD43, and CD45) have been described as Gal-1 ligands, most of them related to the induction of apoptosis of activated T-cells [115]. Several studies have demonstrated that recombinant Gal-1 suppresses T helper (Th)1 and Th17 responses [116] and promotes Foxp3(forkhead box 3)+CD25+CD4+ regulatory T cell differentiation and proliferation [117,118]. In addition, Gal-1 treatment skews the Th1 response toward a Th2 response by upregulating interleukin expression. The study of Grigorian and co-workers [119] showed that T cell receptor, CD45 and cytotoxic T-lymphocyte-associated protein 4 surface concentration and membrane localization are controlled by the Gal-glycoprotein lattice, thereby negatively regulating T-cell growth throughout the growth cycle. We also demonstrated that keratinocytes seeded on decellularized ECM matrices produced by Gal-1-treated dermal fibroblast exhibited epidermal stem-like cell phenotype [5], but recombinant Gal-1 treatment did not stimulate the corneal epithelial wound closure rate in mice [120]. Furthermore, skin wound treatment with Gal-1 resulted in significantly improved contraction rate in a rat model [5]. The molecular mechanism beyond the wound repair improvement was attributed to the activation of Gal-1/neuropilin-1/Smad3 (mothers against decapentaplegic homolog 3)/NOX4 (nicotinamide adenine dinucleotide phosphate oxidase 4) pathway in myofibroblasts that was observed in both healthy and diabetic mice [121]. In squamous cell carcinomas of the head and neck, up-regulation of Gal-1 was significantly correlated to the presence of smooth muscle actin-positive CAFs. Furthermore, significant correlations of several poor-prognosis transcripts (mitogen-activated protein kinase kinase kinase 2 (MAP3K2), tripartite motif containing 23 (TRIM23), protein tyrosine phosphatase-like protein (PTPLAD1), fused in sarkoma interacting serine-arginine-rich protein 1 (FUSIP1), solute carrier family 25 member 40 (SLC25A40), and spindlin 1 (SPIN1)) were detected together with the Gal-1 in CAFs [11]. This data provides new insights into the significance of presence of myofibroblasts in squamous cell carcinoma and points on remarkable similarity to a healing skin wound.

Moreover, other Gals may also contribute to blunt anti-tumor immunity. For example, Gal-3 appears to be a key molecule produced by tumor microenvironment support cells including mesenchymal stromal cells (MSC) to suppress immune surveillance by killing T cells and interfering with NK (natural killer) cell function and by supporting metastasis [122]. In this context in patients who have advanced metastatic melanoma, a clinical trial has been started where a combination of Gal-3 inhibitor (galactoarabino-rhamnogalacturonate GR-MD-02) with ipilimumab is tested. Preliminary results are expected in March 2018 [123]. On the other hand, genetic deletion of Gal-3 did not alter gross wound healing kinetics even though it resulted in delayed re-epithelialization [124]. More importantly, Galectin-3 treatment accelerated re-epithelialization of wounds in Gal-3(+/+) mice but, surprisingly, not in the Gal-3(−/−) mice. Of note, Gal-7 accelerated re-epithelialization of wounds in both Gal-3(−/−) and Gal-3(+/+) mice [120]. In addition to Gal-3, Gal-9 has also been implicated in regulating immune responses by controlling T cell survival [125] and driving the expansion of FoxP3+ Tregs [126].

Angiogenesis, cell adhesion, cell motility, and cell invasion are four important steps of the metastatic process, in which Gals plays a prominent role. They influence stimulation of molecules that affect cell adhesion and cytokines that are essential for tumor metastasis. Over the past few years, increasing evidence has revealed that the pan-carcinoma-associated Thomsen-Friedenreich (TF) antigen is a natural ligand of the galactoside-binding galectins and the oncofetal TF-galectin interaction influences a number of key steps in cancer progression and metastasis [127]. Gals have also been linked to key cellular processes of angiogenesis cascade like endothelial cell adhesion, migration, sprouting, and tube formation [107]. Multiple Gal-1 binding partners have been identified, including integrins such as α7β1 and α5β1, ECM components such as fibronectin and laminin, cytosolic proteins [128]. Moreover, Gal-1 can directly bind to neropilin-1 (NRP1) on endothelial cells, and promote the NRP1/VEGFR-2-mediated signaling pathway [129,130]. Not only cancer- and endothelium-derived Gals induce angiogenesis, CAF-derived Gal-1 increases VEGF expression and enhances VEGFR2 phosphorylation in endothelial cells [131]. Gal-3 also makes a significant contribution to VEGF-A-mediated angiogenesis, facilitating VEGF-R2 plasma membrane retention and phosphorylation [132].

## 7. Conclusions

Data shown in previous subsections clearly demonstrate remarkable similarities between the tumor and wound microenvironments. In this context, tumor stroma exerts several morphological and functional similarities to the granulation tissue [4,7,55]. For example, a basal/squamous cell carcinoma reminds in many aspects the caricature of a healing skin wound.

**Figure 2:**
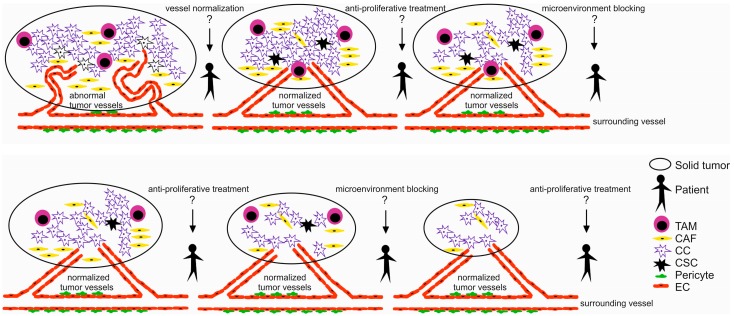
Proper scheduling of solid tumor treatment? Anti-cancer therapy should involve combination of anti-angiogenic (vessel normalization), anti-TME (eliminating cancer stem and progenitor cells) and anti-proliferative therapies (eliminating quickly dividing cancer cells). However, an optimal scheduling of combination therapies is still not known since a complex treatment strategy could result in an improved patient survival (tumor-associated macrophage (TAM), cancer-associated fibroblast (CAF), cancer cell (CC), cancer stem cell (CSC), endothelial cell (EC).

Components of the ECM, growth factors, cytokines/chemokines, and galectins are potent modulators of cancer growth and spreading. However, single molecule therapy demonstrated in many cases only limited clinical efficiency. *Extremis malis extrema remedia*, thus the synergistically acting signaling pathway need to be inhibited to decrease the metastatic capacity of cancer cells and thereby improve patient outcomes. Combination therapy may also reduce drug resistance and attenuate the likelihood of relapse. From this point of view, tailor made manipulation of cancer stroma together with a complex antineoplastic treatment involving all crucial steps of tumor growth (Figure 2) can have important therapeutic consequences. Furthermore, further studies need to focus on the optimal scheduling of combination therapies, which is still not well known and seems to be rather underrated [133]. For example, bevacizumab decreased tumor perfusion, which results in hypoxia and decreased delivery of the drug that was administered in a combination therapy [134] and may lead to a more aggressive tumor growth [135]. This observation highlights the importance of drug scheduling [136] and shows that the administration of anti-angiogenic agents may be considered after the cytotoxic drugs [133]. Similarly, combinations of currently used conventional chemotherapeutics and drugs modulating the TME should also be carefully investigated for optimal scheduling.

Other crucial aspects regulated by the TME and also by tumor cells is the modulation of endogenous antioxidant levels which may be a determining factor for the sensitivity of certain tumors to various chemotherapeutic agents. Therefore, novel drugs modulating anti-oxidative enzymes (e.g., peroxiredoxins) may thus be targets of anti-cancer therapy [137,138]. In relationship to treatment scheduling and combination therapy, it is important to highlight that the regulation of intracellular antioxidant concentration is a “double-edged sword”: on the one hand, enhanced antioxidant activity represents an advantageous protection of the cells from reactive oxygen species whereas, on the other hand, the depletion of antioxidants represents an important strategy to sensitize cancer cells to chemotherapy [139].

Better understanding of cancer cell-stroma interaction can help to improve wound healing by supporting granulation tissue formation and process of reepithelization of extensive and chronic wounds. It has been well demonstrated that topical administration of growth factors may also be a promising strategy in the treatment of chronic wounds. However, administration of a single molecule may not be sufficient for optimal wound treatment since the expression profiles of different factors changes in time and phase of healing. Furthermore, depending on conditions, the same growth factor may activate different signal transduction pathways leading to various cellular responses [140]. From this point of view, sophisticated spatio-temporal controlled delivering systems of growth factors and other signaling molecules with proper treatment scheduling need to be introduced into clinical practice to activate crucial regenerative pathways [141]. Finally, prevention of hypertrophic scars and the formation of keloids may also be a great challenge in this context.

## Figures and Tables

**Table 1:** Comparison of wound healing and squamous/basal cell carcinoma (extracellular matrix (ECM), epithelial to mesenchymal transition (EMT); granulation tissue (GT)) [4,5,6,7].

Event	Wound	Squamous/Basal Cell Cancer
Infiltration by leukocytes	Wound bed, GT/Transitory	Stroma and between cancer cells/Continuous
Accumulation of fibroblasts	GT/Transitory	Stroma/Continuous
Production of ECM		
New capillaries formation		
Myofibroblast formation		
Secretion of proteases from fibroblasts/myofibroblasts	ECM of GT remodelation/Transitory	Stroma remodelation/Continuous
Proliferation of epithelial cells	Reepithelisation/Transitory	Tumor growth/Continuous
EMT	Reepithelisation/Transitory	Locally aggressive growth and metastazing/Continuous

**Table 2:** Overview of currently tested drugs modulating the Tumor Microenvironment (TME).

TME Targets	Strategy	Target + Drugs (Examples)		References
ECM	Inhibition of ECM degradation	MMP inhibitors	Andecaliximab (GS-5745) (anti-MMP-9 monoclonal antibody)	[18]
			Neovastat (*shark cartilage extract AE-941*)	[19]
Growth factors and signalling pathways	Inhibiotion of kinases and kinase receptor activity	Inhibition of kinases and kinase receptor activity	Genistein (protein-tyrosine kinase inhibitor, antioxidant)	[20]
			Plitidepsin 171 (VEGF and VEGFR1 inhibitor, marine invertebrate compound)	
CAFs	Direct targeting of CAFs	FAP-α antibodies	Sibrotuzumab	[21]
			Lu-labeled ESC11; Lu-labeled ESC14	[22]
			Vaccines targeting FAPα	[23]
	CAF-epithelial interaction	HGF-Met signalling	NK4 (HGF antagonist)	[24]
			YYB-101 (monoclonal anti-HGF antibody)	[25]
		NF-κB and STAT3 signaling pathway	EC-70124 (multikinase inhibitor)	[26]
		CXCL12/SDF-1	NOX-A12 (L-stereoisomer RNA aptamer (Spiegelmer))	[27]
		CXCR4	BL-8040 (CXCR4 inhibitor)	[28]
			Plerixafor (CXCR4 antagonist)	[29]
		PDGF-R	Nilotinib (PDGF-R tyrosine kinase inhibitor)	[30]
			Olaratumab (IMC-3G3) (anti-PDGFR-α monoclonal antibody	[31]
			Crenolanib (inhibitor of receptor tyrosine kinases PDGFRα, -β; FLT3)	[32]
		TGF-β ligand inhibitors	Fresolimumab (GC1008) (human anti-TGF-β monoclonal antibody)	[33]
		TGF-β receptor inhibitor	Galunisertib TGF-βRI (TGF-beta receptor I kinase inhibitor)	[34]
	CAF-ECM interaction	Hyaluronan	rHuPH20 (recombinant human hyaluronidase enzyme)	[35]
	CAF-endothelial interaction	PDGF-B	E10030 (Fovista) RNA-based anti-PDGFR aptamer	[36]
	CAF—inflammatory immune cell interactions	IL-6	Siltuximab (anti-IL-6 monoclonal antibody)	[37]
		TNF	Inflinximab and Etanercept (TNF inhibitors)	[38]
Angiogenesis	Growth factors	Bevacizumab (VEGF-A antibody); Aflibercept (chimeric soluble receptor); VEGF-trap; Thalidomide; Lenalidomide; IMC-18F1 (VEGFR-1 signaling); Ramucirumab (VEGFR-2 signaling)		[39,40,41]
	Small molecules tyrosine kinase inhibitors	Sunitinib; Sorafenib; Pazopanib; Axitinib; Vandetanib; Regorafenib; Cabozantinib; Motesanib; Cediranib; Tivozanib		[42]
	Intergrin inhibitors	MEDI-522 (Vitaxin); Cilengitide (EMD 121974); Volociximab (chimeric monoclonal antibody)		[43,44]
	mTOR	Everolimus		[45]
	Human antiangiogenic factors	Endostatin		[46]
		Thrombospondin-1		[47]
	Angiopoietin	Trebananib AGM 386 (angiopoietin-1/-2-neutralizing peptibody)		[48]
Immune system	CSF-1	RG7155 (monoclonal antibody against CSF-1 receptor activation)		[49]
	CTLA-4	Ipilimumab (CTLA-4 monoclonal antibody)		[50]
Galectins	Galectin-3	GR-MD-02		[51]

Abbreviations: matrix metalloproteinase (MMP); interleukin 6 (IL-6); vascular endothelial growth factor (VEGF); VEGF receptor (VEGFR); cancer-associated fibroblast (CAF); fibroblast activation protein (FAP); colony stimulating factor (CSF); cytotoxic T-lymphocyte-associated protein 4 (CTLA-4); mechanistic target of rapamycin (mTOR); transforming growth factor beta (TGF-β); platelet derived growth factor (PDGF); PDGF receptor (PDGFR); chemokine receptor type 4 (CXCR4); hepatocyte growth factor (HGF); hepatocyte growth factor receptor (Met); tumor necrosis factor (TNF); chemokine (C-X-C motif) ligand 12 (CXCL12); stromal cell-derived factor 1 (SDF-1).
